# Arachidonic acid metabolism in health and disease

**DOI:** 10.1002/mco2.363

**Published:** 2023-09-20

**Authors:** Yiran Zhang, Yingxiang Liu, Jin Sun, Wei Zhang, Zheng Guo, Qiong Ma

**Affiliations:** ^1^ Department of Orthopedic Surgery Orthopedic Oncology Institute The Second Affiliated Hospital of Air Force Medical University Xi'an China; ^2^ Department of Pathology The Second Affiliated Hospital of Air Force Medical University Xi'an China

**Keywords:** arachidonic acid, bioactive lipid metabolites, organ homeostasis, targeted therapy

## Abstract

Arachidonic acid (AA), an n‐6 essential fatty acid, is a major component of mammalian cells and can be released by phospholipase A2. Accumulating evidence indicates that AA plays essential biochemical roles, as it is the direct precursor of bioactive lipid metabolites of eicosanoids such as prostaglandins, leukotrienes, and epoxyeicosatrienoic acid obtained from three distinct enzymatic metabolic pathways: the cyclooxygenase pathway, lipoxygenase pathway, and cytochrome P450 pathway. AA metabolism is involved not only in cell differentiation, tissue development, and organ function but also in the progression of diseases, such as hepatic fibrosis, neurodegeneration, obesity, diabetes, and cancers. These eicosanoids are generally considered proinflammatory molecules, as they can trigger oxidative stress and stimulate the immune response. Therefore, interventions in AA metabolic pathways are effective ways to manage inflammatory‐related diseases in the clinic. Currently, inhibitors targeting enzymes related to AA metabolic pathways are an important area of drug discovery. Moreover, many advances have also been made in clinical studies of AA metabolic inhibitors in combination with chemotherapy and immunotherapy. Herein, we review the discovery of AA and focus on AA metabolism in relation to health and diseases. Furthermore, inhibitors targeting AA metabolism are summarized, and potential clinical applications are discussed.

## Graphical abstract

Arachidonic acid (AA) is released from phospholipids in the cell membrane by cytosolic phospholipase A2 (cPLA2) and catabolized into different metabolites mainly through cyclooxygenase (COX), lipoxygenase (LOX), and cytochrome P450 (CYP‐450) metabolic pathways. Arachidonic acid metabolites play important roles in cell fates, such as proliferation, differentiation, and apoptosis, as well as in fibrosis and inflammation caused by tissue injury. Accumulating evidence has proven that AA and its metabolites function in organ development and diseases, such as cardiovascular development, kidney development, liver development, skeletal development, diabetes, obesity, and cancers. Additionally, arachidonic acid metabolic pathways are important targets for drug development.

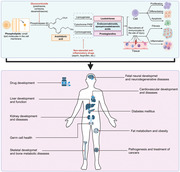

## 1 INTRODUCTION

Arachidonic acid (AA), also known as eicosatetraenoic acid (C_20:4_, ω−6), has been found to be an important polyunsaturated fatty acid present in human tissue that is usually esterified as glycerolipids or glycerophospholipids to maintain the structure and function of the cell membrane.^1^ AA was first named by J. Lewkowitsch in 1913; however, the exact structure was not elucidated until the 1940s.^2^, ^3^, ^4^ It is not only important for normal cellular membrane fluidity but is also a substrate for numerous enzymatic transformations that form biologically active lipid mediators such as prostaglandins (PGs), leukotrienes (LTs), epoxyeicosatetraenoic acids (EETs), and endocannabinoids (ECs).^5^

**FIGURE 1:**
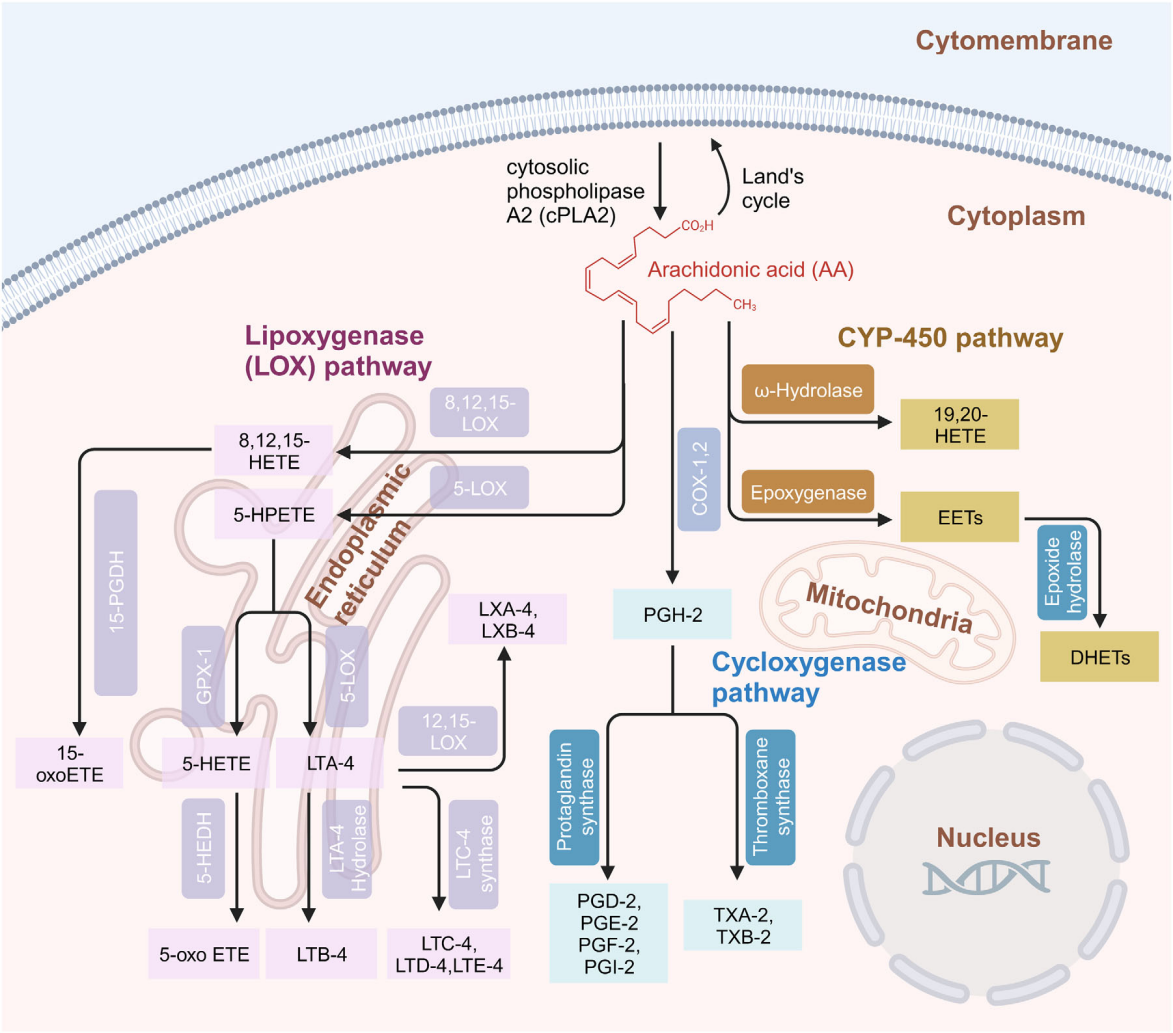
Metabolites and enzymes involved in AA metabolism. AA is released from phospholipids by cPLA2, and it can be reincorporated into phospholipids (Land's cycle) or can be enzymatically changed into active metabolites mainly through three metabolic pathways involving COX, CYP‐450, or LOX. cPLA2, cytosolic phospholipase. COX, cyclooxygenase. The enzymes involved in AA metabolism are mainly found in the cytoplasm, endoplasmic reticulum, mitochondria and nuclear membrane of the cell. CYP‐450, cytochrome P450; LOX, lipoxygenase; 15‐HPDG, 15‐hydroxyprostaglandin dehydrogenase; HETE, hydroxyeicosatetraenoic acid; HPETE, hydroperoxyeicosatetraenoic acid; LTA‐4 hydrolase, leukotriene A(4) hydrolase; LCT‐4 synthase, leukotriene C(4) synthase; 5‐oxo‐ETE, 5‐oxoeicosatetraenoic acid; PGE‐2, prostaglandin E‐2; PGF‐2, prostaglandin F‐2; PGD‐2, prostaglandin D‐2; PGI‐2, prostaglandin I‐2; TXA‐2, thromboxane A‐2; TXB‐2, thromboxane B‐2; EETs, epoxyeicosatrienoic acids; DHETs, dihydroxyeicosatrienoic acids; 5‐HEDH, 5‐hydroxyeicosanoid dehydrogenase; GPX‐1, glutathione peroxidase 1.

These active mediators of AA are mainly metabolized through three canonical metabolic pathways regulated by different enzymes can be detected in the cytoplasm, endoplasmic reticulum, mitochondria, and nuclear membrane of the cell (Figure 1).^6^, ^7^, ^8^, ^9^, ^10^, ^11^, ^12^ The enzymes involved in the cyclooxygenase (COX) pathway are COX‐1 and COX‐2, along with downstream enzymes that mediate the production of prostaglandins (PGH‐2, PGE‐2, PGD‐2, PGF‐2, PGI‐2, TXA‐2, and TXB‐2). The lipoxygenase (LOX) pathway consists of 5‐LOX, 8‐LOX, 12‐LOX, and 15‐LOX and their products, namely, LTA‐4, LTB‐4, LTC‐4, LTD‐4, and LTE‐4, lipoxins (LXA‐4 and LXB‐4), and hydroperoxyeicosatetraenoic acid (HPETE). Another one is the cytochrome P450 (CYP‐450) pathway which involves two enzymes, CYP‐450 epoxygenase (EPO) and CYP‐450 ω‐hydroxylase, giving rise to EETs and 19, 20‐hydroxyeicosatetraenoic acid (19, 20‐HETE), respectively. Another important enzyme involved in AA metabolism is acyl‐coenzyme A synthetase long‐chain isoform 4 (ACSL‐4), which is an isozyme that catalyzes AA into glycerophospholipids.^13^, ^14^ Additionally, AA can also undergo nonenzymatic reactions, and studies have shown that autoxidation of AA is triggered by reactive oxygen species (ROS)‐ and reactive nitrogen species (RNS)‐induced oxidative stress.^15^

AA metabolism usually varies from cell to cell according to different factors; consequently, the levels and types of biosynthesized eicosanoids will vary based on different situations. For instance, the state of cells, that is, whether stimulated or in the resting phase, will cause this variation.^9^, ^16^ Some studies have shown that when COX‐2 and 5‐LOX gene expression is inhibited, there is compensatory upregulation of COX‐1.^17^, ^18^ PGE‐2 and LTB‐4, which are two major metabolites of the COX‐2 and 5‐LOX pathways, have opposite effects on the inflammatory response. Both LTB‐4 and LTD‐4 oppose the suppressive effect of PGE‐2 on the phagocytosis of alveolar macrophages in innate immune functions.^19^ Therefore, dual inhibitors of COX‐2 and 5‐LOX have been developed to exert synergistic anti‐inflammatory and antitumor effects.^20^, ^21^ Additionally, there is cross‐talk between different AA metabolic pathways. Inhibition of soluble epoxide hydrolase (sEH), a major enzyme that degrades EETs produced by CYP‐450 enzymes, leads to repression of COX‐2 gene expression and alleviates the inflammation caused by lipopolysaccharide (LPS),^22^ and exogenous 11,12‐EET could suppress the synthesis of PGE‐2 in rat monocytes.^23^ Accumulating evidence has indicated the importance of AA metabolites in the regulation of life activities.

This paper focuses on the role of AA metabolites during tissue and organ development and related diseases to provide a comprehensive understanding of the relationship between AA metabolites and human health. Furthermore, approved drugs and potential therapeutic chemicals targeting AA metabolism are summarized, which will be helpful to determine the application of AA metabolic pathway inhibitors in the clinic and to guide the development of targeted drugs in the future.

## 2 AA METABOLISM IN HUMAN HEALTH AND DISEASES

### 2.1 AA metabolism in bone development and bone diseases

#### 2.1.1 AA metabolites are involved in the regulation of molecular signaling in bone metabolism

**FIGURE 2:**
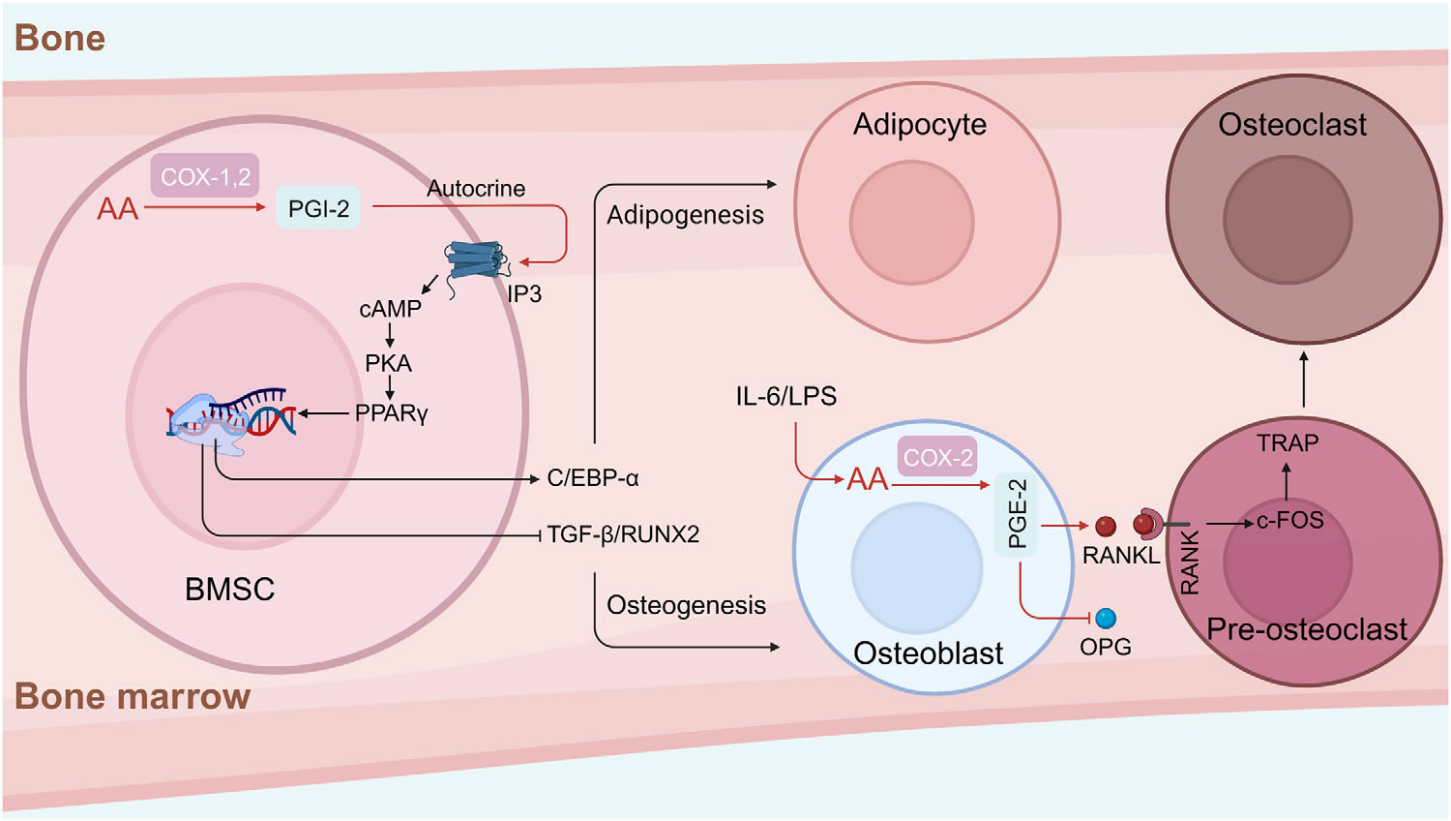
Regulation of bone marrow stem cells (BMSCs) differentiation by AA metabolites. AA metabolites, such as PGI‐2 and PGE‐2, facilitate adipocyte differentiation by promoting the expression of PPARγ and suppress osteoblast differentiation by inhibiting the expression of TGF‐β and RUNX2. In the inflammatory state, COX‐2 and PGE‐2 are induced by IL‐6 and LPS in osteoblasts, resulting in the downregulation of OPG and the upregulation of RANKL, which ultimately promote osteoclast differentiation through the RANK–cFOS–TRAP pathway. PGI‐2, prostaglandin I‐2; IL‐6, interleukin‐6; LPS, lipopolysaccharide; RANK, receptor activator of nuclear factor kappa‐B; RANKL, ligand to receptor activator of NFkB ligand; c‐FOS, cellular oncogene fos; TRAP, tartrate‐resistant acid phosphatase; PGE‐2, prostaglandin E2; PPARγ, peroxisome proliferator‐activated receptor; OPG, osteoprotegerin.

Many studies have shown that AA metabolites are involved in bone development and bone diseases. Bone development is the result of a series of synchronous events, including osteogenesis and bone resorption. Osteoblasts, osteoclasts and chondrocytes are critical for bone homeostasis.^24^ AA signaling is important for the differentiation and function of osteoblasts, osteoclasts and chondrocytes. Ca^2+^ is required for osteoblastogenesis, and AA causes a concentration‐dependent increase in the intracellular Ca^2+^ concentration due to the action of the COX metabolites—PGE‐1 and PGE‐2.^25^ Osteoblasts are differentiated from bone marrow‐derived mesenchymal stem cells (BMSCs), which are subject to subtle gene expression regulation. Peroxisome proliferator‐activated receptor gamma (PPARγ) is a key regulator that governs the differentiation of BMSCs into adipocytes through cyclic adenosine monophosphate‐protein kinase A system (cAMP–PKA) signaling via the regulation of PGI‐2.^26^, ^27^, ^28^ In the inflammatory state, osteoblasts are stimulated by inflammatory factors, such as interleukin‐6 (IL‐6) and LPS, resulting in the activation of the AA metabolic pathway, causing an imbalance in osteoprotegerin/receptor activator of nuclear factor kappa‐B (OPG/RANK) and affecting bone homeostasis due to the proliferation and differentiation of osteoclasts (Figure 2).^29^, ^30^

Moreover, treatment of BMSCs with a COX‐2 inhibitor suppressed the osteogenic genes encoding runx2, alkaline phosphatase, and osteocalcin.^31^, ^32^ The effect of AA on bone formation is also reflected in the regulation of cytokine and growth factors expression. Insulin‐like growth factor‐1 (IGF‐1) is a crucial growth factor that regulates bone mass, enhances osteoblast differentiation, and promotes bone formation. PGE‐2 has been shown to stimulate IGF‐1 and its binding proteins. Studies have suggested that AA might influence bone formation and resorption by regulating the synthesis and action of IGF‐1 and IGF binding proteins.^33^, ^34^ In vitro studies have shown that the bone resorption activity of PGE‐2 mediated by receptor activator of nuclear factor‐κb ligand (RANKL) is critical for the induction of osteoclast formation. Moreover, bone resorption stimulated by inflammation involves PGE‐2 production.^35^, ^36^ Interestingly, PGE‐2 also promotes osteogenesis by stimulating osteoblast proliferation and differentiation.^37^ These findings indicate that the nature of PGE‐2 in bone remodeling is multifaceted. LTB‐4 promotes peripheral blood mononuclear cell differentiation into osteoclasts in a RANKL‐dependent manner,^38^ and it reduces mineralized nodule formation and alkaline phophatase (ALP) activity in primary osteoblasts.^39^ LTD‐4, another leukotriene metabolite, significantly increases the expression of p53, p21, and plasminogen activator inhibitor‐1 (PAI‐1), as well as the activity of senescence‐associated β‐galactosidase (SA‐β‐Gal), with commensurate reduction in SIRT1 expression, resulting in the senescence of osteoblasts.^40^ Relatively few studies have been focused on the AA metabolite‐mediated differentiation and development of chondrocytes by AA metabolites. One study showed that PGE‐2 inhibited the expression of the differentiation‐related genes in chondrocytes, such as collagen‐X (col‐X), vascular endothelial growth factor (VEGF), matrix metalloproteinase‐13 (MMP‐13), and ALP, in a dose‐dependent manner, resulting in inhibited chondrocyte maturation.^41^

#### 2.1.2 AA metabolites play essential roles in bone metabolic diseases

The effect of AA metabolites on osteoblasts and osteoclasts is of great importance to bone mineral density and bone mass.^42^ A condition closely related to bone disease is osteoporosis. In vivo studies showed that administration of AA into ovariectomized mice decreased the bone mineral density and weakened biomechanical functions by reducing the mineral apposition rate and impairing the microstructure of trabecular bone. The underlying mechanism was described as AA inducing an increase in the content of serum PGE‐2 and causing a marked increase in the expression of tumor necrosis factor associated factor‐6 (TRAF6), nuclear factor‐kappa b (NF‐κB), c‐fos proto‐oncogene (c‐fos), and nuclear factor of activated T‐cells, cytoplasmic 1 (NFATc1), which leads to a decrease in the OPG/RANKL ratio.^43^ Endocannabinoids produced during AA metabolism via the fatty acid amide hydrolase (FAAH) pathway play important roles in the regulation of bone homeostasis. Administration of FAAH ameliorated ovariectomy‐induced bone loss in mice by suppressing RANKL through the regulation of IL‐17 signaling.^44^ Lipoxin A4 (LXA‐4) is a metabolic product of AA produced by lipoxidase. A study showed that LXA‐4 reduced ovariectomy‐induced bone loss by inhibiting NF‐κB, activator protein‐1 (AP‐1), and phosphoinositide 3 kinase‐threonine kinase (PI3K‐AKT) activity, as well as p38, extracellular regulated protein kinase (ERK), and c‐Jun N‐terminal kinase (JNK) in the mitogen activated protein kinases (MAPKs) activation. Furthermore, LXA‐4 prevented the production of ROS and the expression of osteoclast‐specific genes, resulting in inhibited osteoclastogenesis.^45^

AA metabolite signaling not only contributes to bone metabolism but also exerts regulatory effects on the occurrence of inflammatory bone diseases such as osteoarthritis, rheumatoid arthritis and synovitis. Ye et al.^46^ discovered that AA‐regulated calcium signaling in T cells from rheumatoid arthritis patients led to increased activity of the AA‐regulated calcium‐selective (ARC) channel and the phosphorylation of components in the T‐cell receptor signaling cascade, which resulted in the promotion of synovial inflammation. Nonsteroidal anti‐inflammatory drugs (NSAIDs) targeting COX‐2 have been widely used in the clinic to treat arthritis.^47^, ^48^ Considering the side effects of COX‐2 inhibitors as treatments for arthritis,^49^ many efforts have been made to develop safer drugs. Jiang et al.^50^ found that PGE‐2 mediated cell migration and osteoclastogenesis via its prostaglandin E receptor 2 (EP2) and prostaglandin E receptor 4 (EP4) receptors; therefore, drugs aimed at EP4 showed better selectivity in treating osteoarthritis.

Considering the role of AA metabolic pathways in the regulation of bone homeostasis and diseases, such as osteoarthritis, targeting related metabolites could be effective in modulating skeletal development and osteoarthritic diseases, thus overcoming the adverse effects of currently used clinical drugs such as glucocorticoids.^51^

### 2.2 AA metabolism in the physiological development, and function and in diseases of the liver and kidney

#### 2.2.1 AA metabolism in liver development and diseases

Studies focused on the expression of CYP‐450 EPO family members (CYP2C8, CYP2C9, CYP2C19, and CYP2J2) in human embryonic/fetal tissues have shown that CYP2C8, CYP2C9, and CYP2C19 is initially expressed in the liver in prenatal week 5 and remains steadily expressed until the 20th week. The expression of CYP2J2 is also initiated in the liver in the 5th prenatal week, peaks in the 12th week and then gradually decreased to the normal level.^52^ AA is an essential component of the cell membrane. Jakobsson et al. utilized radioactive phospholipids to identify the types of fatty acids in the hepatocellular membrane. AA constitutes approximately 10‐30% of the cell membrane composition.^53^, ^54^ AA is not only a main component of the hepatocyte membrane but also participates in the proliferation and differentiation of hepatocytes. Transforming growth factor‐beta (TGF‐β) is a multifunctional cytokine that is pivotal in the regulation of hepatic cell proliferation, differentiation, and migration.^55^, ^56^, ^57^ Han et al.^58^ discovered that TGF‐β regulated the growth of primary and transformed hepatocytes through the concurrent activation of Smad and phosphorylation of cPLA_2_, which indicated that AA metabolic signaling may be indirectly involved in the regulation of hepatocyte differentiation and development. Additionally, intracellular Ca^2+^ signaling is essential for cell development.^59^ Notably AA inhibited the store‐operated Ca^2+^ flow but did not activate Ca^2+^‐permeable channels in rat liver cells.^60^

AA is not the only important factor in the liver, liver function and health also depend on feedback mechanisms regulated by AA metabolites. Experiments have shown that the FAAH level is decreased in murine models of liver fibrosis, which leads to increased ROS levels and hepatocellular injury.^61^ In addition, 2‐arachidonoyl glycol (2‐AG) has been reported to selectively induce hepatic cell apoptosis and cause liver dysfunction.^62^ In liver cirrhosis, the level of COX‐2 but not that of COX‐1 was significantly increased, and the use of selective COX‐2 inhibitors in patients with liver cirrhosis was beneficial in reducing inflammation and preventing malignant transformation.^63^ However, in transgenic animal models, COX‐2 did not appear to be the cause of liver fibrosis or cirrhosis because hepatocytes overexpressing human COX‐2 and COX‐2 knockout mice showed the same level of hepatic injury as wild type mice in response to CCl_4_.^64^ In addition, the role of 5‐LOX, 15‐LOX‐1, and 15‐LOX‐2 in alcohol‐induced liver cirrhosis has also been studied. The results showed that increased plasma HETE concentrations were in line with upregulated 5‐ and 15‐LOX‐1 and 15‐LOX‐2 mRNA in liver samples from cirrhosis patients.^65^ AA metabolic signaling plays important roles in liver cancer. COX‐2/PGE‐2 signaling promotes the survival of liver cancer cells. Studies have shown that when hepatocellular carcinoma cell (HCC) lines were treated with selective COX‐2 inhibitors, the proliferation of liver cancer cells was suppressed.^66^, ^67^ Targeted treatment of HCC revealed that the combined use of COX‐2 inhibitors with sorafenib showed a synergistic inhibitory effect on tumor growth and angiogenesis in mice bearing HCC xenografts.^68^ 5‐LOX is another AA metabolic signaling pathway that has been studied in depth in liver cancer. Expression of the 5‐LOX protein was upregulated both in HCC cell lines and in patient tumors. 5‐LOX and its metabolite LTB‐4 have been shown to participate in the metastasis of HCC cells. A 5‐LOX inhibitor but not a COX inhibitor reduced the number of metastatic foci. Moreover, the administration of an antagonist of the LTB‐4 receptor reduced the number of lung metastasis foci.^69^

#### 2.2.2 AA metabolism in kidney development and diseases

In contrast to that in the liver, the expression pattern of the PGE‐2 synthetic system in the kidney has been associated with postnatal nephrogenesis. The transient induction of the mRNA and protein expression of microsomal PGE synthase‐1 has been observed between postnatal days 4 (P4) and 8 (P8) during the first 10 days after birth, and the protein levels of both COX‐1 and COX‐2 reached their highest levels on P8.^70^ CYP2J5 expression was the most abundant in the kidney and was lower in the liver. CYP2J5 was expressed before birth and reached maximal levels in the kidney 2−4 weeks of age. However, CYP2J5 is not expressed in the fetus and is first expressed in 1 week of the early postnatal period and remains relatively constant in the liver.^71^ Although these studies suggest that the temporal and spatial expression of these AA metabolites is precisely regulated, the underlying mechanisms remain largely unknown. Recently, AA and its metabolites have been shown to be baroreceptors that transduce changes in mechanical pressures via nuclear mechanotransduction mechanisms to change gene expression and renin cell function.^72^

AA metabolic signaling also plays important roles in maintaining kidney function. Reportedly, LOX and CYP‐450 alter renal blood flow and the glomerular filtration rate.^73^ Studies based on renal blood flow have shown that although COX‐2 is expressed in the macula densa and plays an important role in renin secretion, COX metabolites are not critical components for the renal blood flow regulatory pathway. Similarly, no glomerular LOX metabolites have been shown to contribute to renal blood flow regulation. On the other hand, the critical role of CYP metabolites in renal blood flow has been demonstrated, and 20‐HETE is a key component of the afferent arteriolar autoregulatory response.^74^ The roles of COX metabolites, LOX metabolites, and CYP metabolites involved in regulating renal blood flow can be distinguished by selective AA metabolic signaling inhibitors or agonists.^75^ Increased afferent arteriolar constriction to increasing perfusion pressure were greatly attenuated by the selective CYP hydroxylase inhibitor.^76^ The 20‐HETE synthesis inhibitor HET0016 and the antagonist 6,15‐20‐HEDE completely blocked the myogenic response in afferent arterioles.^77^, ^78^ The mechanisms underlying the roles of CYP and LOX in glomerular filtration mainly involve K^+^ and Ca^2+^ channels, cyclic guanosine monophosphate (cGMP) and cAMP.^73^ For example, the LOX metabolite 12(S)‐HETE has been shown to influence renal microvessels and glomerular mesangial cells through L‐type Ca^2+^ channels.^79^ 20‐HETE inhibits Na^+^‐K^+^‐ATPase activity by enhancing the protein kinase C (PKC) pathway activation.^80^ Furthermore, 20‐HETE downregulates Na^+^‐K^+^‐ATPase α1 expression via the ubiquitin‒proteasome pathway, and phosphorylated Na^+^‐K^+^‐ATPase α1 is a prerequisite for ubiquitination.^81^ CYP and LOX metabolites also interact with the nitric oxide (NO) synthase and ROS pathways, which are involved in the glomerular filtration barrier.^82^ 12‐/15‐LOX catalyze the depletion of NO and prevent NO activation of cGMP, and knocking out of 12‐/15‐LOX results in increased eNOS expression.^83^ Because of the important role of AA metabolic signaling in renal blood flow and glomerular permeability, the occurrence and development of kidney diseases such as nephritis, renal fibrosis, and renal cancer are closely related to AA metabolite levels.^84^ Thus, intervening in AA metabolism, such as inhibiting the 5‐LOX pathway,^85^ COX‐2 pathway,^86^ or CYP‐450 pathway,^87^ is a promising approach to regulate renal function. All of the abovementioned studies indicate that targeting the regulation of AA metabolites and related signaling pathways is a potential approach to the clinical treatment of liver and kidney diseases.

Although AA metabolic pathways have their own expression patterns in the liver and kidney, metabolites play essential roles in the development of the liver and kidney. AA metabolic pathways have similar effects on liver and kidney disease, mainly in terms of oxidative stress, fibrosis, and modulation of the vascular microenvironment. The understanding of these common factors will assist in the development of drugs for the treatment of liver and kidney disease.

### 2.3 AA metabolism in neurodevelopment and nervous system disease

#### 2.3.1 Effects of AA on fetal neurodevelopment

**FIGURE 3:**
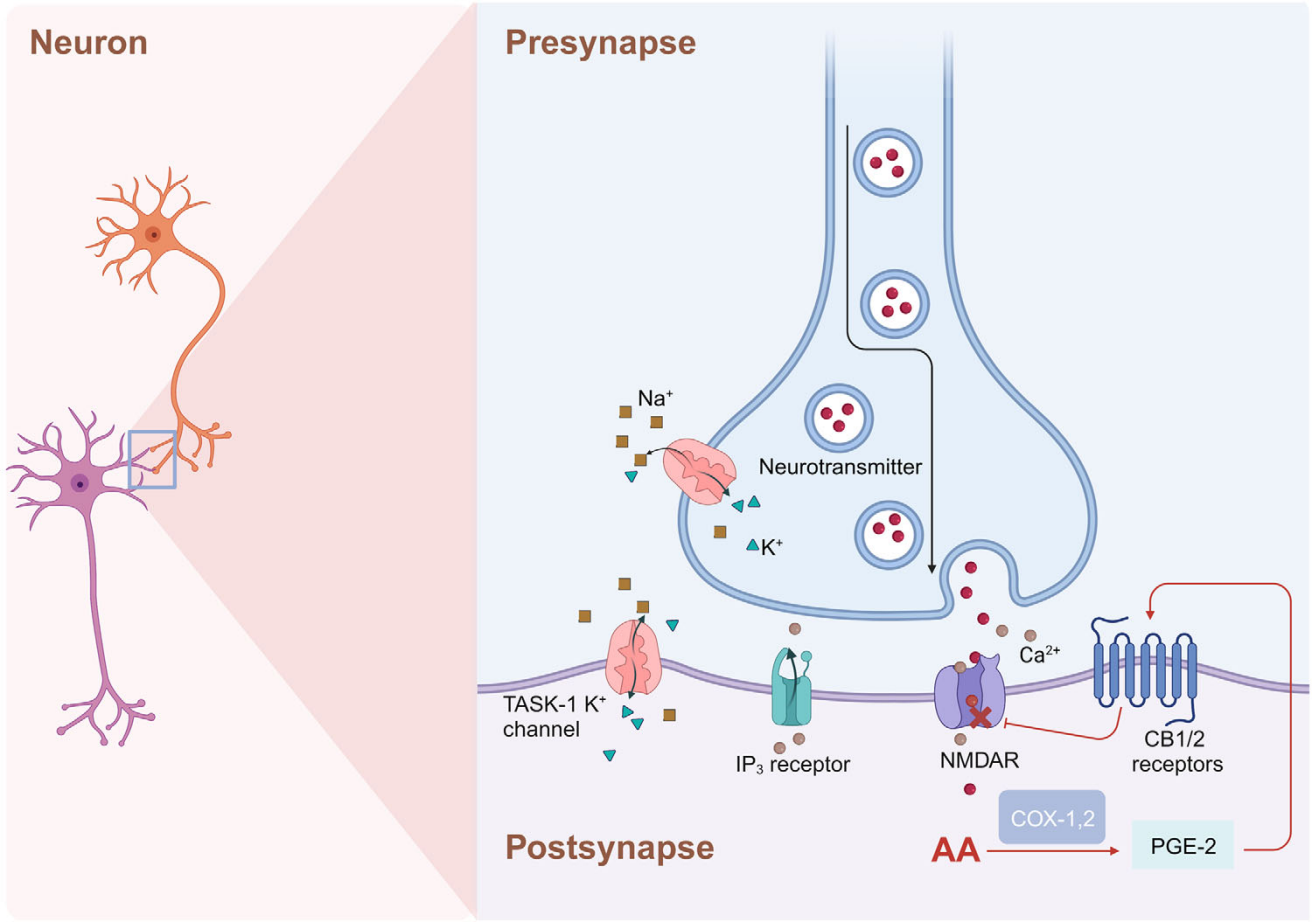
Roles of AA metabolites in the control of synaptic transmission. Potassium influx and sodium efflux via the TASK pathway, postsynaptic IP3 receptor‐mediated Ca^2+^ release from internal stores, and AA metabolites could reduce N‐methyl‐D‐aspartic receptor‐induced calcium and neurotransmitter (like d‐serine) influx via CB1‐mediated closing of voltage‐sensitive calcium channels in the brain. CB, cannabinoid receptor. NMDAR, N‐methyl‐d‐aspartic acid receptor; TASK, TWIK‐related acid‐sensitive K; IP_3_ receptor, inositol 1,4,5‐trisphosphate receptors.

Neurodevelopment is a complex physiological event that requires an adequate nutrient supply. Among key nutrients, the long‐chain polyunsaturated fatty acids (PUFAs) docosahexaenoic acid 22:6n‐3 (DHA) and AA 20:4n‐6 (ARA) have been proven to be required for fetal neurodevelopment.^88^, ^89^ Compared with those of DHA, the roles of AA in neurodevelopment are not well characterized. The accumulation of AA in the fetal brain, particularly within the cerebral cortex mainly occurs in the middle and terminal stages of pregnancy.^90^, ^91^ Data showed that AA accounted for 12% of the fatty acids in the whole brain,^92^ and supplementation with AA in early life benefits to cognition in early and middle childhood.^93^, ^94^ Studies have shown that AA metabolites affect the transmission of synaptic signals mainly through the G protein‐coupled receptors (GPCRs) cannabiniod receptors (CB1 and CB2), which are highly expressed in the hippocampus, basal ganglia, cerebellum, cortex, thalamus, amygdala, and olfactory bulb. Furthermore, non‐GPCR molecular targets of AA metabolites have been discovered, such as the membrane cation channels known as TWIK‐related acid‐sensitive K (TASK‐1 K^+^) channels, T‐type Ca^2+^ channels, and vanilloid type 1 receptor (VR1).^95^, ^96^, ^97^ In long‐term depression state, postsynaptic IP3 receptor‐mediated Ca^2+^ release from internal stores, postsynaptic eicosanoids synthesis, and activation of CB1 receptors possibly via release of the gliotransmitter d‐serine.^98^ Notably, the underlying mechanism of AA metabolites regulation of Ca^2+^ influx between neuron synapses may rely on reduced N‐methyl‐D‐aspartic acid receptor (NMDAR)‐induced calcium influx via CB1‐mediated closure of voltage‐sensitive calcium channels in the brain (Figure 3).^99^

However, the content of AA in the brain is unaffected by postnatal dietary supplementation, which is different from that of DHA.^100^ The reason for this difference between AA and DHA is likely caused by the distinctive gene expression pattern of the fatty acid desaturase (*FADS*‐gene) cluster and differences in sensitivity to dietary supplementation.^101^, ^102^ The roles of AA metabolic signaling in neurodevelopment have been studied via genetically altered mouse models.^103^ COX‐2 knockout mice exhibited decreased susceptibility to ischemia/reperfusion brain injury and 1‐methyl‐4‐phenyl‐1,2,3,6‐tetrahydropyridine (MPTP)‐induced brain injury.^104^ COX‐2 plays an important role in cholinergic signaling in the brain, as indicated by the abrogation of AA incorporation in response to arecoline in COX‐2 knockout mice, suggesting that COX‐2 is required for muscarinic receptor activation.^105^ Data from PGE‐2 receptor‐EP‐1 knockout mice showed that the EP1 receptor mediated neurotoxicity after NMDA treatment, oxygen/glucose deprivation, or focal ischemia.^106^ However, another PGE‐2 receptor, EP‐2, exerted a protective effect against NMDA‐induced neurotoxicity in the hippocampus.^107^ 12‐/15‐LOX knockout mice were protected against neuronal cell death and oxidative stress caused by transient focal ischemia.^108^ These studies indicate that the AA metabolic signaling pathway is involved mainly in mediating oxidative stress and neurotoxicity.

#### 2.3.2 AA metabolism in neurodegenerative diseases

In nervous system diseases, the relationship between AA and its metabolites and degenerative brain diseases, such as Alzheimer's disease and Parkinson's disease, has been widely reported.^109^ The level of intracellular free AA and the balance between the release and incorporation of membrane phospholipid enzymes played critical roles in neuroinflammation and synaptic dysfunction in mice with Alzheimer's disease before amyloid plaques and neurofibrillary tangles developed.^110^ COX‐2 is overexpressed in the cortex and hippocampus of Alzheimer's disease patients.^111^, ^112^ 5‐LOX protein expression is also upregulated in Alzheimer's disease patients, and immunoreactivity induced using 5‐LOX amino terminus‐directed antibodies was lacking in neurons but abundant in neurofibrillary tangles, neuritic plaques, and glia.^113^ Increasing evidence has proven that 5‐LOX is involved in Aβ peptide formation and deposition. Chu and Pratico reported that 5‐LOX modulated Aβ peptide generation by activating cAMP‐response element‐binding protein (CREB) and promoting γ‐secretase complex expression at the transcriptional level in Tg2576 and 3xTg mice and N2A‐APPswe cells.^114^, ^115^ In addition, 5‐LOX overexpression resulted in the activation of cyclin‐dependent kinase 5 (cdk5), which was followed by the significant elevation in the tau phosphorylation rate, which suggests another important mechanism underlying Alzheimer's disease.^116^ Genetic deletion and inhibition of 5‐LOX inhibited cdk5‐dependent tau phosphorylation and glial reactions and relieved synaptic dysfunction and memory impairment.^117^, ^118^ Additionally, abnormal activation of 5‐LOX has been found in the pathological progression of cerebral ischemia. A direct connection between 5‐LOX products (LTC‐4 and LTD‐4) and ischemic brain damage was reported in bilateral common carotid occlusion‐induced transient forebrain ischemia in gerbils.^119^ In summary, although further studies are still required to understand the roles of AA metabolites in regulating neurological function, the inhibition of AA metabolic signaling pathways is a promising therapeutic strategy for brain dysfunction and neurodegenerative diseases.

As an important component of fatty acids in the brain, the role of AA in brain development and cognitive function remains a mystery. In some psychiatric diseases, such as bipolar disorder, a decreased turnover of AA and its metabolites was found, instead of docosahexaenoic acid.^120^ In addition, as more evidence accumulates, interventions in AA metabolic pathways may be of great importance for the alleviation of neurodegenerative diseases.

### 2.4 AA metabolism in cardiovascular system development and disease

#### 2.4.1 AA metabolism in vascular tone and blood pressure

**FIGURE 4:**
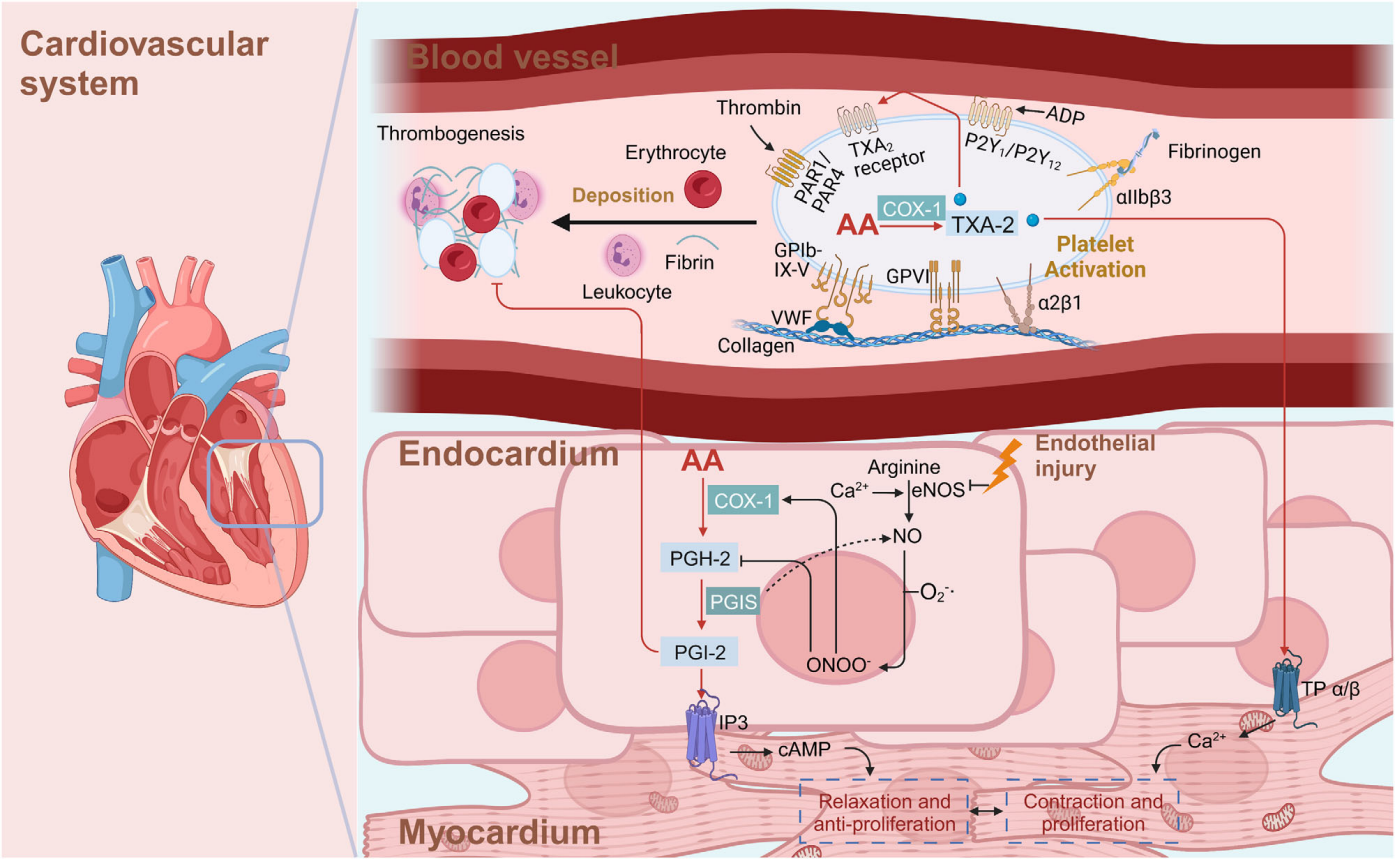
Roles of AA metabolites in the cardiovascular system. On the one hand, vascular endothelial injury reduces the release of endothelial protective molecules (PGI2 and NO), triggering the aggregation of platelets, red blood cells, and fibrin deposits and leading to thrombosis. On the other hand, platelet aggregation is induced through the interaction between collagens and platelets via self‐activation by TXA‐2 caused by COX‐1. Furthermore, relaxation and contraction of myocardial cells are regulated by cAMP and Ca^2+^ signaling triggered by PGI‐2 and TXA‐2. ADP, adenosine diphosphate; PAR1/4, protease‐activated receptor 1/4; VWF, von Willebrand factor; PGIS, prostacyclin synthetase; PGI‐2, prostaglandin I‐2; TXA_2_, thromboxane; COX‐1, cyclooxygenase 1; TXA‐2, thromboxane synthase‐2; cAMP, cyclic adenosine monophosphate.

AA and its metabolites play pivotal roles in cardiovascular function (Figure 4). They function as vasodilators or vasoconstrictors and modulate vasodilation under pathological and physiological conditions.^121^, ^122^ Some blood vessels carry EPO, and AA can be metabolized to epoxides through EPO. Notably, that 5,6‐epoxides exhibit vasodilation‐inducing effects. In addition, AA and its metabolites are considered endothelium‐derived relaxing factors, which causes endothelium‐dependent vasodilation. COX is another important enzyme in AA metabolism and is highly active in platelets. When activated by collagen or thromboxane, platelets release ADP and 5‐HT, increase the synthesis of thromboxanes (TXs), and thus cause platelet aggregation.^123^, ^124^ EPO metabolites inhibit COX activity in platelets, reduce the TX production rate, and thus inhibit platelet aggregation. In this way, EPO metabolites show synergistic effects with other antiplatelet aggregation factors, such as PGD‐2 and PGI‐2, and their levels are balanced with the levels of platelet aggregation‐promoting factors like adenosine diphosphate (ADP), 5‐hydroxtryptamine (5‐HT), and TXs.

Prostacyclin (PGI‐2) is the main product of AA metabolism in the blood vessel wall and is the most effective endogenous inhibitor of platelet aggregation. Investigations with animals and humans have clearly shown the endothelial thromboresistance and atheroprotection conferred by vascular COX‐2‐derived PGI‐2.^5^ Honda et al.^125^ found that oral administration of selexipag, a recently approved, orally available and selective PGI‐2 receptor agonist, significantly reduced right ventricular systolic pressure in Sprague‒Dawley SuHx rats.^126^

TXA‐2 is a major metabolite of AA in platelets and vascular endothelial cells and promotes platelet aggregation and induces thrombosis. TXA‐2 promotes the dissociation of calcium ions in the dense tubule system, causes the contraction of dense bodies, induces the release of ADP and 5‐HT, and cause the aggregation of nearby platelets.^127^ TXA‐2 in activated platelets contributes to primary hemostasis and atherothrombosis in animal and human models. Additionally, TP receptors are activated by TXA‐2 and induce vasoconstriction. Under normal physiological conditions, the levels of TXA‐2 and PGI‐2 in circulating blood are relatively balanced, which is important maintaining smooth blood circulation. An imbalance in TXA‐2 and PGI‐2 levels may lead to thrombosis and tissue ischemia. When thrombosis occurs, the TXA‐2 level is usually increased or the PGI‐2 level is decreased.^128^

LTs constitute a group of inflammatory substances produced via AA metabolism through the 5‐LOX pathway, increasing the incidence of myocardial infarction (MI), stroke, atherosclerosis, and aortic aneurysm.^129^ LTB‐4 reportedly stimulated the release of contraction factors and NO by activating the LT receptor of aortic endothelial cells and induce endothelium‐dependent vasoconstriction, thus promoting atherosclerosis, which is also closely related to the formation of atherothrombosis through NF‐κB signaling.^130^, ^131^ LTC‐4, LTD‐4, LTE‐4, and LTF‐4 belong to the CysTL family and are involved in the proliferation and migration of vascular smooth muscle cells.^132^, ^133^, ^134^

EETs and HETEs participate in the regulation of vascular endothelial cells and smooth muscle cells proliferation, migration and apoptosis and function as endogenous vasodilators to mediate the contraction of blood vessels. The normally functioning EETs and HETEs play important roles in maintaining the stability of vascular system functions and angiogenesis. However, abnormal EET and HETE metabolism may readily lead to the occurrence of vascular disorders, such as hypertension.^135^

EETs are metabolites of CYP‐450 epoxidases, and they include 5,6‐, 8,9‐, 11,12‐, and 14,15‐EETs, which are endothelium cell‐derived hyperpolarizing factors and play important protective roles in the cardiovascular system.^136^ EETs are released from the vascular endothelium and exert effects on smooth muscle cells under endothelial cells. Generally, they activate potassium calcium channels in the cell membrane, thereby hyperpolarizing the cell membrane and leading to the relaxation of smooth muscle cells.^137^ Moreover, EETs contribute to vasomotor tone control of endothelial cells by activating calcium‐activated potassium channels, endothelial NO synthase transcription and MAPK, PI3K/AKT, and PKC signaling.^138^, ^139^

HETEs are important endogenous factors that facilitate the depolarization of smooth muscle cells and maintain the contraction of smooth muscle cells by inhibiting the activation of many calcium channels on the surface of vascular smooth muscle. Thus, HETEs increase the intracellular calcium concentration.

In 1989, Escalante et al.^140^ first reported the vasoconstrictive effects of 20‐HETE in the rat aorta. 20‐HETE activated PKC, MAPK, and src‐type tyrosine kinase, which elevated the cytosolic Ca^2+^ level and increased the Ca^2+^ entry rate through specific channels.^141^ 20‐HETE also maintained the phosphorylation rate of myosin light chains through Rho kinase and increased the Ca^2+^‐related contraction rate.^142^, ^143^

Studies in animals and humans demonstrated that deficient 20‐HETE biosynthesis in tubules and increased 20‐HETE levels in the vasculature contribute to hypertension by sensitizing the vasculature to constriction‐inducing stimuli, potentiating vascular inflammation, and causing endothelial dysfunction.^142^, ^144^, ^145^ Another study of vascular calcification in mice demonstrated that the levels of multiple metabolites of AA were significantly increased in calcified aortas; the abundant metabolites included 12‐HETE, 11‐HETE, and 15‐HETE, of which the most abundant metabolite was 12‐HETE. A specific inhibitor of the metabolic enzyme arachidonic 15‐LOX (ALOX15) significantly reduced the plasma 12‐HETE level, promoted calcium deposition in the aortic arch and increased the calcium level in blood vessels.^146^

Blockade of 20‐HETE by the nonspecific and specific inhibitors 1‐ABT and HET‐0016 could lead to reduced mean arterial pressure by 20–30 mmHg in old female rats with spontaneous hypertension.^147^, ^148^ Probable G‐protein coupled receptor 75 (GPR75), a receptor of 20‐HETE, is a novel target for hypertension treatment.^149^

#### 2.4.2 AA metabolism in heart failure

Ischemic myocardial injury is the most common cause of heart failure. In recent years, many studies have suggested that EETs and 20‐HETE play an important role in preventing ischemic myocardial injury.^150^, ^151^, ^152^ In rat and mouse models of ischemia‒reperfusion injury, low concentrations of 11,12‐EET perfusion treatment before myocardial ischemia reduced the reperfusion‐induced MI area and increased cardiac function.^153^, ^154^, ^155^ In canine and rabbit models of myocardial ischemia/reperfusion, the level of 20‐HETE in the coronary artery was significantly increased. When the generation of 20‐HETE was inhibited, cardiac function after myocardial ischemia/reperfusion injury was improved.^156^, ^157^ These above results show that 20‐HETE aggravates cardiac function inhibition after myocardial ischemia/reperfusion injury, while EETs alleviate cardiac dysfunction caused by ischemia/reperfusion injury.

Heart failure involves a persistent systemic inflammatory reaction. The cascade of inflammatory cytokines reactions plays an important role in the occurrence and development of heart failure.^158^ 20‐HETE gradually activates nicotinamide adenine dinucleotide phosphate (NADPH) oxidase to produce more ROS through the MAPK/ERK pathway, promote inflammatory reactions and aggravate myocardial cell damage.^159^ EETs and their metabolites can activate PPARγ and endogenous growth factor receptor (EGFR), inhibit inflammation, reduce myocardial cell damage, and prevent the occurrence and development of heart failure.^160^, ^161^, ^162^

Heart failure can also be caused by cardiomyocyte apoptosis. Myocardial cell apoptosis leads to weakened cardiac contractile function, which causes a decline in cardiac function. EETs promote the expression of antiapoptotic proteins and reduce the activity of apoptotic effector proteins.^163^, ^164^ 20‐HETE causes the release of a large amount of ROS from vascular endothelial cells and directly acts on DNA to induce cell apoptosis.^165^ Moreover, it enhances cell membrane permeability, promotes Ca^2+^ influx, and causes intracellular calcium overload to induce cell apoptosis. In addition, the accumulation of ROS exerts a regulatory on the expression of apoptosis‐related genes and changes the levels of the proteins they encode, inducing apoptosis. Bao et al.^166^ found that after 20‐HETE administration, the survival rate of neonatal rat cardiomyocytes decreased, and the number of cells in the early and late stages of apoptosis increased markedly.

Another risk factor for heart failure is cardiac hypertrophy. 20‐HETE activates the calcineurin/nuclear factor of activated T‐cell signaling pathway, a key pathway in the cardiac hypertrophy signaling pathway, thereby promoting cardiac hypertrophy.^167^ In contrast, EETs effectively inhibit cardiac hypertrophy, promote ventricular remodeling, and delay heart failure by regulating the expression of MMPs or activating the EGFR/PI3K/Akt/CREB signaling pathway to promote atrial natriuretic peptide secretion.^168^, ^169^

In general, AA metabolites show high biological activity, and balance in the effects of AA metabolites is very important. These metabolites are especially important to research on vascular movement and blood coagulation, atherosclerosis, hypertension, and antithrombotic drugs and the treatment of heart failure.

### 2.5 AA metabolism in obesity and diabetes

#### 2.5.1 AA metabolism in obesity

Obesity is an independent risk factor for systemic diseases. A study assessing the fatty acids in adipose tissues of children in Crete and Cyprus demonstrated that the subcutaneous AA levels in all of the obese children were in the highest quartile.^170^ A clear correlation between body weight and the regulation of anandamide level was found. The level of circulating 2‐AG in obese patients was significantly increased and positively correlated with body mass index and abdominal adiposity.^171^ Ailhaud et al.^27^ found that AA stimulated adipogenesis and weight gain through its effect on PGI‐2 pathway by upregulating CCAAT/enhancer‐binding protein B (C/EBPB) or activating PPARγ and PPARβ/PPARδ.

Lipoxin is a biologically active substance synthesized from AA in a reaction catalyzed by LOXs, mainly LXA‐4 and LXB‐4. LXA‐4 inhibits IκBα degradation and NF‐κB translocation to attenuate the inflammatory the M1 macrophage phenotype acquired in the context of obesity.^172^ Börgeson et al.^173^ found that LXA‐4 and LX analogs reduced obesity‐induced inflammatory reaction in adipose tissue, changed the ratio of M1/M2 macrophages in adipose tissues, and decreased obesity‐induced autophagic flux. In addition, lipoxins also reduce obesity‐induced liver disease and chronic kidney disease. Börgeson and Sharma^174^ reported that lipoxins restored insulin sensitivity and inhibited renal fibrosis by regulating leukocyte infiltration and promoting the elimination of inflammation in visceral adipose tissue. These results indicate that lipoxins exhibit therapeutic potential in obesity‐induced pathological damage.

Obesity is one of the causes of diabetes, especially type 2 diabetes. Obese patients usually have more visceral fat. This accumulation of visceral fat can lead to insulin resistance and reduce the action of insulin effect, leading to failed glucose utilization in the body and a gradual increase the plasma glucose level, which leads to diabetes. The specific role of AA metabolism in diabetes mellitus (DM) is described in the following subsection.

#### 2.5.2 AA metabolism in DM

DM is a metabolic disease characterized by hyperglycemia caused by defective insulin secretion or insulin dysfunction. As a PUFA, AA in diabetes patients plays a contradictory role. On the one hand, the AA level in the circulatory system of diabetes patients is low, and exogenous intake of AA effectively attenuates the abnormal lipid metabolism and insulin resistance characteristic of DM.^175^ On the other hand, a significant increase in the AA level in the circulatory system of diabetes patients inhibits insulin secretion, promoting the occurrence and development of DM.^176^

Reportedly, the AA metabolic pathway in type 2 diabetes model mice is abnormal. The expression levels of fatty acid synthase, phospholipase A2, COX‐2, and LOX‐5 and the levels of fasting blood glucose, insulin, AA, and related metabolites in the liver of diabetic mice are significantly increased, while the expression of carnitine palmitoyltransferase 1A and CYP‐450 family 4A are significantly reduced.

Studies on AA metabolites showed that different metabolites contribute differentially to insulin resistance, depending on the cell and tissue type. In white adipose tissues, PGE‐2 promotes adipogenesis and induces glycogen decomposition and gluconeogenesis, thereby reducing the insulin resistance of adipocytes.^177^, ^178^ PGE‐2 also enhances the dysfunction and destruction of pancreatic islet β‐cells and hinder insulin secretion. In contrast, PGI‐2 increases the insulin sensitivity of pancreatic cells. Recently, scholars found that a higher level of PGF‐2α in diabetic mice was related to gluconeogenesis in the liver, and PGF‐2α was also the main factor associated with fasting hyperglycemia in type 2 diabetes patients.^179^ In addition, the 12‐/15‐LOX enzyme induces the production of various HPETEs. These HPETEs interact with PPARα and PPARβ and are involved in cytokine‐mediated damage to β‐cells in pancreatic islets. 12‐/15‐LOX‐knockout mice showed resistance to the development of diabetes to some extent.^180^ Similarly, LTs produced by HETEs also showed an inhibitory effect on insulin secretion.^181^, ^182^ LTB‐4 is vitally important for the recruitment and activation of B2 lymphocytes in adipose tissue, which may contribute to insulin resistance after a high‐fat diet.^183^ In contrast, 20‐HETE and EET could protect pancreatic β‐cells from apoptosis and promote the secretion of insulin.^184^

Reportedly, human and rat pancreatic islet cells can produce EETs and stimulate the release of insulin and glucagon. 5,6‐EETs play important roles in the excitation–coupling process of pancreatic islet β‐cells, which increase insulin secretion by activating the concentration of Ca^2+^.^185^ 8,9‐EETs, 11,12‐EETs, and 14,15‐EETs stimulate the secretion of glucagon.^186^ Increasing the level of EETs tighten the control of blood glucose levels by increasing insulin sensitivity. In addition, Xu et al.^187^ found that CYP2J3/EETs may reduce insulin resistance via the PI3K/AKT and MAPK pathways or membrane translocation of GLUT‐4 through the HO‐1/adiponectin pathway. Diabetes has also been associated with the increased expression of sEH, which is the enzyme critical for the degradation of EETs. In a diabetic mouse model, the deletion of the sEH gene led to an increase in insulin sensitivity and antiapoptotic effects on pancreatic islet cells.^188^ The therapeutic potential of sEH inhibitors is currently being evaluated in clinical trials.

In conclusion, AA metabolites play different roles in the pathogenesis of both obesity and diabetes. Studies into AA metabolism and related enzyme pathways may lead to the identification of new targets for clinical treatment.

### 2.6 AA metabolism in germ cell development

Reproduction is closely related to the metabolic state of the organism.^189^ AA and LA are very important during pregnancy.^190^ These factors are closely related to ovulation, menstruation, pregnancy, and childbirth and the occurrence of the physiological inflammatory response.^191^, ^192^

AA metabolism is essential for oocyte maturation. The maturation of oocyte meiosis is one of the important physiological requirements for ovulation and fertility. Cyclic adenosine phosphate, the protein kinase A and protein kinase C pathways, and AAs, especially PGE‐2 and steroids, are believed to be key factors in the regulation of mammalian oocyte maturation. It is important to note that the release of AA is strictly regulated by follicle‐stimulating hormone and luteinizing hormone during oogenesis. Developmental reproduction is closely related to the metabolic state of the organism.^193^, ^194^ Studies have shown that supplementation with maternal fatty acids can significantly promote the embryo elongation of bovine embryos, and in bovine granulosa cells, lower doses of AA increased the survival of bovine granulosa cells, whereas higher doses of AA suppressed survival.^195^ While lower doses of AA induced the accumulation of lipid droplets in granulosa cells, the higher dose of AA inhibited lipid accumulation, and AA increased the abundances of fatty acid binding protein 3 (FABP3), CD36, and long chain fatty acid transport protein 1 (SLC27A1) mRNA. Higher doses of AA decreased the secretion of 17β‐estradiol (E2) and increased the secretion of progesterone (P4) accompanied by downregulation of the mRNA expression of CYP19A1, FSHR, HSD3B1, and STAR in granulosa cells.^196^ The offspring of diabetic rats usually show abnormal neural development, but maternal diabetes supplementation with AA can effectively improve the neurodevelopmental abnormalities in the offspring; moreover, dietary ω‐3 and ω‐6 PUFAs restored fertility in young and adult fads2‐deficient mice.^197^, ^198^ Polycystic ovarian syndrome (PCOS) is a common reproductive endocrine disorder in women of reproductive age. A study found that PCOS patients had higher levels of AA than those of normal control subjects. AA in follicular fluid induces oxidative stress in a human ovarian granulosa tumor cell line and upregulates the expression of growth differentiation factor 15 (GDF15).^199^ In follicles, lower levels of n‐3 PUFAs are more responsive to ovarian stimulation.^200^ Moreover, the increase in lecithin in follicular fluid indicated diminished early embryonic development.^201^

The fatty acid composition of follicular fluid is related to oocyte maturation and quality. A study showed that the fatty acid composition in follicles is related to the developmental potential of oocytes up to the blastocyst stage.^202^ The fatty acid composition in follicular fluid can reflect the oocyte cytoplasm under assisted reproductive therapy (ART).^203^ In addition, in embryos established in vitro that lacked a normal early development environment, such as a reduced normal fatty acid supply, showed developmental defects, and abnormal AA metabolism led to abnormal DNA methylation of these embryos.^204^

AA is also important for male reproductive health.^205^ ALOX1 mediates 4‐hydroxynonenal‐induced protein damage in male germ cells, so specific inhibition targeting ALOX15 could be used to protect human sperm from oxidative stress.^206^, ^207^

### 2.7 AA metabolism in inflammation

AA metabolic network is the main network that produces inflammatory mediators and induces inflammation. AA and its metabolites are widely involved in immune and inflammatory reactions in vivo. Free fatty acids, including unsaturated and saturated fatty acids, are widely involved in the inflammatory response,^208^, ^209^, ^210^ which can link metabolism with immunity and inflammation.^191^, ^211^ The correlation between DHA and AA levels in preterm infants is closely related to early systemic inflammation.^212^ To control inflammation, there are multiple ways of perturbing the AA metabolic network.^213^ In response to irritation or cell damage, AA is released from the cell membrane by the action of phospholipase A2. Once released, AA produces a large amount of metabolites through the action of COX, LOX, and CYP‐450. CYP‐450‐derived eicosanoids compounds are widely involved in the inflammation associated with liver disease.^214^ CYP‐450‐mediated AA metabolism may influence and affect inflammation associated with cardiac hypertrophy.^215^

AA metabolites are generally considered inflammatory bioactive lipids and can promote kidney inflammation.^11^, ^216^, ^217^ AA metabolites, including prostaglandins, maintain homeostasis and mediate disease‐causing mechanisms, including inflammatory responses.^218^ At the same time, studies have found that supplementation with AA oil in diabetic rats have anti‐inflammatory and hypoglycemic effects.^219^

#### 2.7.1 AA metabolism regulates signaling pathways involved in inflammation

AA and its metabolites have strong biological activity and can participate in the inflammatory response by activating various signaling pathways. Abnormal fatty acid metabolism can amplify the inflammatory response of infection by regulating the p38 MAPK signaling pathway.^220^, ^221^ Calcium signaling pathways in T cells promote synovial inflammation in patients with rheumatoid arthritis.^222^ The phenotype of alternatively activated macrophages (M2‐type macrophages) differs from that of classically activated macrophages (M1‐type macrophages). Both phenotypes are important for the innate and adaptive immune systems. AA and its metabolites were also involved in the macrophage phenotype switching. This effect is mainly dependent on the regulation of PPARγ mediated oxidative phosphorylation.^223^

#### 2.7.2 AA metabolism regulates inflammation at different levels

AA can regulate inflammatory responses independently of its metabolites. In cultured cardiomyocytes and macrophages, AA directly regulates inflammatory responses by regulating toll‐like receptor 4 (TLR4) activity. AA inhibits the formation of the TLR4 complex and accessory protein induced by saturated fatty acids. This effect is mainly achieved through direct binding of AA to the TLR4 coreceptor myeloid differentiation factor 2 and the blocking of TLR4 proinflammatory signaling pathway activation by saturated fatty acids.^224^

Disorders of the intestinal flora can cause disorders of AA metabolism.^225^ This disorder further exacerbates the inflammation associated with atherosclerosis.^226^ Peritoneal macrophages (rpMACs) produce inflammatory lipid mediators by secreting PUFAs and AA in response to infection or tissue damage. Chronic Acsl4 deficiency in rpMACs reduces the incorporation of AA into phospholipids, thereby reducing lipid mediator synthesis and inflammation.^227^, ^228^ Intervention of the AA pathway in mouse macrophages prevents endotoxin‐induced inflammation, which is mediated by aldose reductase.^229^, ^230^ Obesity is associated with low levels of chronic inflammation, in which AA cascades play a key role. AA treatment resulted in significant downregulation of proinflammatory markers and COX pathways. AA treatment can effectively reduce adipocyte inflammation induced by a high‐fat diet in obese mice.^231^ AA may influence obesity through the enteric–hypothalamic–adipose–liver axis.^232^ The development of nonalcoholic fatty liver disease is accompanied by inflammation. Early screening indicators are of great significance for the prevention of the disease. Studies have shown that AA can be used as an early detection indicator.^233^ In chronic obstructive pulmonary disease, AA increases inflammation but inhibits extracellular matrix protein expression.^234^ Studies on mouse‐derived pulp tissue during acute inflammation have shown that AA and LA metabolites regulate the expression of catalase.^235^ In addition, air pollution also exacerbates inflammatory progression. Studies have shown that AA‐ and LA‐derived hydroxyl metabolites are associated with air pollution and interact with systemic inflammation in the process of body response to air pollution.^236^

#### 2.7.3 Chinese herbal medicine regulates inflammation through AA metabolism

Chinese herbal medicine can also regulate AA metabolism to play an anti‐inflammatory role. Magnolol is the main active ingredient in Magnolia officinae. Studies have found that the active ingredient in Magnolia officinae interferes with its function mainly by directly inhibiting the activity of enzymes related to AA metabolism, such as COX‐2, or by inhibiting the mRNA and protein expression of COX‐2 to affect the AA metabolic pathway and exert a good anti‐inflammatory effect.^237^ Saikosaponin can improve chronic pelvic inflammatory disease by regulating niacin and niacinamide metabolism and AA metabolism,^238^ and Paeoniflorin can improve COX‐2 expression in the AA pathway.^239^ Inflammation can be controlled by disrupting the body's AA metabolic network in a number of ways.

### 2.8 AA metabolism in cancer

**FIGURE 5:**
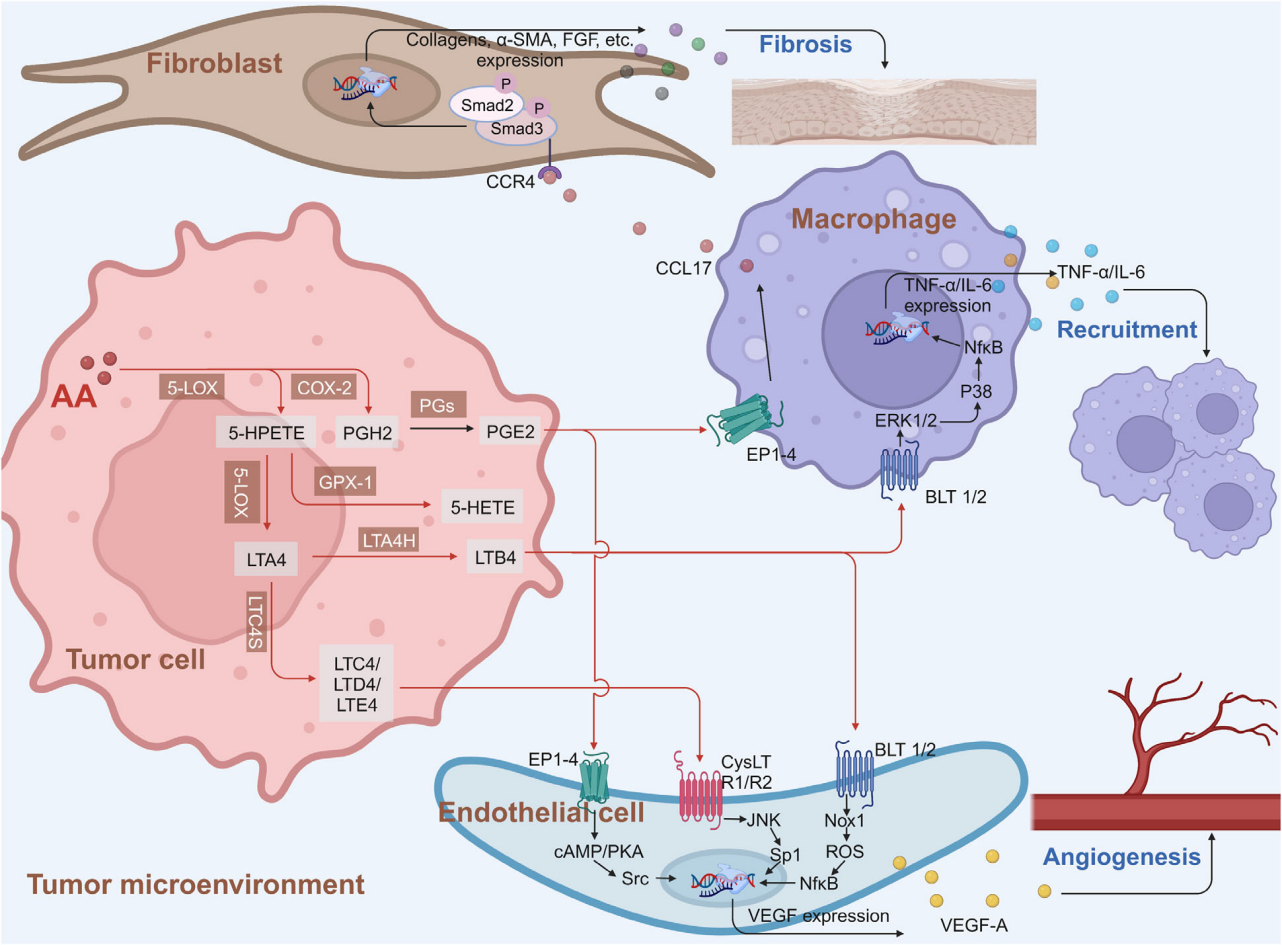
Roles of AA metabolites in the regulation of the tumor microenvironment. AA metabolites act as messengers among tumor cells, fibroblasts, macrophages, and endothelial cells. For example, PGE2 and LTB4, which are derived from the COX‐2 and 5‐LOX AA metabolic pathways, promote the expression of cytokines through BLT 1/2, CysLT R1/R2, or EP 1−4 receptors and lead to the recruitment of macrophages through ERK‐P38 MAPK/NF‐κB signaling, as well as the release of CCL17, which binds to CCR4 and leads to fibrosis via upregulation of collagens, α‐SMA, and FGF. AA metabolites, such as PGE‐2, LTB‐4, and LTC4, can also trigger angiogenesis by regulating the expression of VEGF in endothelial cells. FGF, fibroblast growth factor; VEGF‐A, vascular endothelial growth factor‐A; TNF‐α, tumor necrosis factor‐α; IL‐6, interleukin‐6; BLT 1/2, leukotriene B4 receptor 1/2; CysLT R1/R2, cysteinyl‐leukotriene receptor; EP 1−4, prostaglandin EP receptor 1−4; CCL17, CC chemokine ligand 17; CCR4, CC chemokine receptor 4; P‐Smad 2/3, phosphorylated Smad 2/3; α‐SMA, α‐smooth muscle actin.

Cancer cells undergo metabolic remodeling, which provides the ATP and macromolecules needed for rapid cell growth, division, and survival. AA and its metabolites are associated with the occurrence of a variety of tumors, and the importance of alterations in fatty acid metabolism in cancer cells has attracted much attention.^240^, ^241^, ^242^ In addition to being structural components of the membrane matrix, fatty acids are also important secondary messengers, and different secondary metabolites of fatty acids can participate in the occurrence and development of tumors.^243^, ^244^ We briefly summarized the mechanisms of AA and its metabolism‐related enzymes in regulating key signaling pathways, regulating gene expression, and influencing cell apoptosis (Figure 5).

AA is a polyunsaturated fatty acid that is widely present in mammalian cell membranes. AA is mainly metabolized to hydroxyeicosapentaenoic acid, EET, prostaglandins, and other active metabolites through the CYP‐450, LOX, and COX pathways. A large number of studies based on tumor cell lines have shown that AA and its metabolites promote tumor development by regulating cell carcinogenesis, progression and distinct tumor cell processes, including proliferation, chemotaxis, migration, and apoptosis.^245^, ^246^, ^247^, ^248^

#### 2.8.1 AA metabolism activates tumor‐related cell signaling pathways

AA can regulate tumor proliferation through a variety of signaling pathways. In MDA‐MB‐231 breast cancer cells, AA and its metabolites are involved in cell migration by activating FAK. In the process of malignant transformation, AA passes through PLA2α. Src, ERK1/2, and LOX activity‐dependent pathways promoted GalT I expression in MDA‐MB‐231 breast cancer cells.^249^ AA also activated Akt2 through Src, EGFR, and PI3K and promoted the migration and invasion of MDA‐MB‐231 cells. In addition, AA promoted NF‐κB‐DNA binding activity in an Akt‐dependent manner.^250^ Triple‐negative breast cancer is an aggressive subtype of breast cancer that poses a challenge to treatment because it does not respond to estrogen and progesterone receptor inhibitors. The endogenous AA synthetic pathway, delta 6 desaturase (D6D) activity, and PGE‐2 levels are increased in breast tumors, particularly those of the ER‐ genotype.^251^ Store‐operated Ca^2+^ entry has been implicated in the migration of some cancers. Studies have found that AA regulates Ca^2+^‐selective channels (ARC channels), activates Ca^2+^ entry, and then inhibits tumor invasion. However, in MDA‐MB‐231 cells, AA was found to impair the proliferation and migration capacity of MDA‐MB‐231 cells by activating apoptosis and reducing cell viability. However, these effects are not related to changes in Ca^2+^.^252^ In neuroendocrine cancer cell lines, AA treatment could promote cell migration, and this migration could be inhibited by selective inhibition of AA‐induced Ca^2+^ influx.^253^

#### 2.8.2 AA metabolism regulates tumor‐related gene expression

The expression of AA and its downstream metabolites is regulated by other abnormally activated genes in tumor cells. In pancreatic cancer, untargeted metabolomics analysis revealed that AA and its downstream metabolites are regulated by HDAC5, which inhibits cPLA2 expression by deacetylating GATA1. Knockdown of HDAC5 results in a significant increase in AA and its downstream metabolites. HDAC5 negatively regulates the expression of the gene encoding calcium‐dependent phospholipase A2 (cPLA2), a key enzyme in the formation of AA from phospholipids.^254^ Increased ATF6α expression in prostate cancer (PCa) leads to a CrPC‐like phenotype in PCa cells.^255^ High ATF6α expression enhances Pla2G4A‐mediated AA metabolism and protects tumor cells against iron downregulation to promote the progression of PCa. Therefore, genetic inhibitors targeting ATF6α can promote the apoptosis of iron cells and delay the progression of PCa in tumor cells.^256^

Bone metastasis of PCa is a major challenge in clinical treatment. Studies have shown that some SNPs in AA metabolism genes may influence PCa susceptibility. A case‒control study found that SNPs in the COX‐2, PTGES2, ALOX5, ALOX5AP, and LTA4H genes were associated with PCa susceptibility.^257^, ^258^ Noel Clarke found that AA induces PCa cells to enter the bone marrow, a new finding that may help explain why taking statins, commonly used cholesterol‐lowering drugs, can slow the progression of the disease in some cases. Using PCa cells, AA has been shown to attract PCa cells to bone marrow. When PCa cells were exposed to AA, the researchers found that the tumor cells changed shape, which helped the cells pass through gaps in the surrounding tissue and establish metastases in the bone marrow, but statin administration disrupted the tumor cells’ ability to make cholesterol, stopping the cells from acquiring these properties. These studies show how fatty acids naturally produced by the bone marrow directly interact with the body's cholesterol‐producing system to improve the ability of PCa cells to spread around the body. This information provides important clues as to how PCa patients might benefit from drugs such as statins.^259^

In colon cancer HT‐29 cells, AA inhibits lipogenesis, leading to a lack of sufficient fatty acids to support cell division and subsequently inducing endoplasmic reticulum stress and apoptosis.^260^ Other studies have shown that DHA and AA exert different anticancer activities in colorectal cancer cells in vitro. DHA inhibits the proliferation of HT‐29 cells more than AA, and its effect is mainly through decreasing proteasome granules, while ARA has a significant effect on all six DNA replication helicase granules.^261^, ^262^ Methyl donor restriction in colon cancer has been shown to cause significant changes in fatty acid metabolism. Regulation of fatty acid metabolism by restricting methyl donors can achieve certain therapeutic effects.^263^ In addition to regulating tumor cell survival, AA can attenuate tumor progression by remodeling the tumor microenvironment.

#### 2.8.3 AA metabolism in the tumor microenvironment

The tumor microenvironment is characterized by inflammation and immunosuppression and includes high levels of unsaturated fatty acids. A large number of studies have confirmed that the presence of AA and its metabolites is an important factor promoting tumor metastasis and invasion.^264^ Researchers have identified phospholipase PLA2G2A as the most clinically relevant extracellular AA‐producing enzyme. This finding offers potential treatment options for further diagnosis. AA in the ovarian cancer microenvironment promotes the survival of ovarian cancer cells by disrupting the structure of lipid rafts, destroying janus tyrosine kinase‐signal transducer and activator of transcription (JAK–STAT signaling in macrophages and inhibiting the recognition of immune cells by macrophages.^265^ In addition to the direct effects of AA, its metabolites are also involved in the immunosuppressive effects of the tumor microenvironment.^266^ PGI‐2 released by tumor‐associated fibroblasts was found to promote immunosuppression and metastatic macrophage polarization in the ovarian cancer microenvironment.^267^ AA contributes to an unfavorable clinical outcome of OC by impacting the phenotype of tumor‐associated macrophages via the ASK1–p38δ/α (MAPK13/14) regulatory axis.^268^ Through comprehensive analysis of three databases, the researchers found that the AA metabolism level was positively correlated with the prognosis of breast cancer patients, indicating that AA might promote the immune killing function of the body toward tumor cells, that is, AA metabolism is a cancer suppressor factor for breast cancer.^269^ The team also found a positive correlation between AA metabolism levels in breast cancer and the levels of CD8+ T cells and activated NK cells.

#### 2.8.4 AA metabolism regulates ferroptosis

Iron is an indispensable trace element that can easily become deficient in the human body and is also essential for the proliferation and development of tumor cells. Ferroptosis is an iron‐dependent nonapoptotic cell death mode.^270^ There is growing evidence that ferroptosis may be associated with a variety of pathological conditions, including acute kidney injury, tissue ischemia‒reperfusion injury, neurodegeneration, and cancer.^271^ Recent studies have shown that AA is also related to the mechanism of ferroptosis induction.^272^ The polyunsaturated fatty acid biosynthesis pathway determines susceptibility to ferroptosis in gastric cancer.^273^ Liao et al.^274^ found that T‐cell‐derived interferon (IFN) γ binds to AA to induce ferroptosis in immunogenic tumors. IFNγ alone or single fatty acids failed to induce cell death in the two mouse melanoma lines Yumm5.2 and B16F10 and the human melanoma line A375. However, AA induces effective cell death in all three tumor cell lines by synergistic action with IFN‐γ.^274^

#### 2.8.5 AA metabolism modulates chemotherapy sensitivity

Chemotherapy sensitivity is an important factor that affects the efficacy of tumor treatment. AA can modulate the chemotherapy sensitivity of tumor cells. The protective effect of bleomycin on IMR‐32 cells was further enhanced by exogenous apoptotic pathway activation by AA, and reprogramming of arachidonate metabolism conferred temozolomide resistance to glioblastoma by enhancing mitochondrial fatty acid oxidation activity.^275^, ^276^ Sp1‐regulated PGE2 production activates fatty acid oxidation (FAO) and the tricarboxylic acid cycle (TCA cycle) in mitochondria through EP1 and EP3 receptors, resulting in temozolomide (TMZ) resistance in glioblastoma multiforme (GBM). These results will provide us with a new strategy to attenuate drug resistance or to resensitize recurrent GBM. In malignant mesothelioma, AA drives adaptive responses to chemotherapy‐induced stress. Pemetrexed promoted the release of excess AA from the malignant mesothelioma cell line cPLA2 and activated the NF‐kB signaling pathway. Increased AA mediated the expression regulation of drug resistance‐related genes.^277^

## 3 THERAPEUTIC STRATEGIES TARGETING AA METABOLISM

AA and its metabolites are involved in the occurrence and development of a variety of diseases. Therefore, more AA metabolic targets need to be further explored for use in developing individualized targeted therapy. For diseases caused by different AA metabolic enzymes, we can choose specific inhibitors targeting this metabolic enzyme for combined treatment.

At present, there are a variety of chemosynthetic small‐molecule compounds and active components derived from natural products that can target COX, LOX, and CYP‐450. Zileuton is a potent oral 5‐LOX inhibitor that inhibits the formation of LTB‐4, LTC‐4, LTD‐4, and LTE‐4 and is used to relieve the symptoms of asthma. Zileuton can induce apoptosis and inhibit ferroptosis.^258^ Etoricoxib is a novel selective COX‐2 inhibitor with anti‐inflammatory, antipyretic, analgesic, and potentially antitumor activities.^278^, ^279^ Naringin (naringoside) is a flavonoid glycoside and is the main flavonoid in grapefruit, causing the bitter taste of grapefruit juice. It has antioxidant, lipid lowering, and antitumor activities and exerts an inhibitory effect on CYP‐450 expression.^280^

**FIGURE 6:**
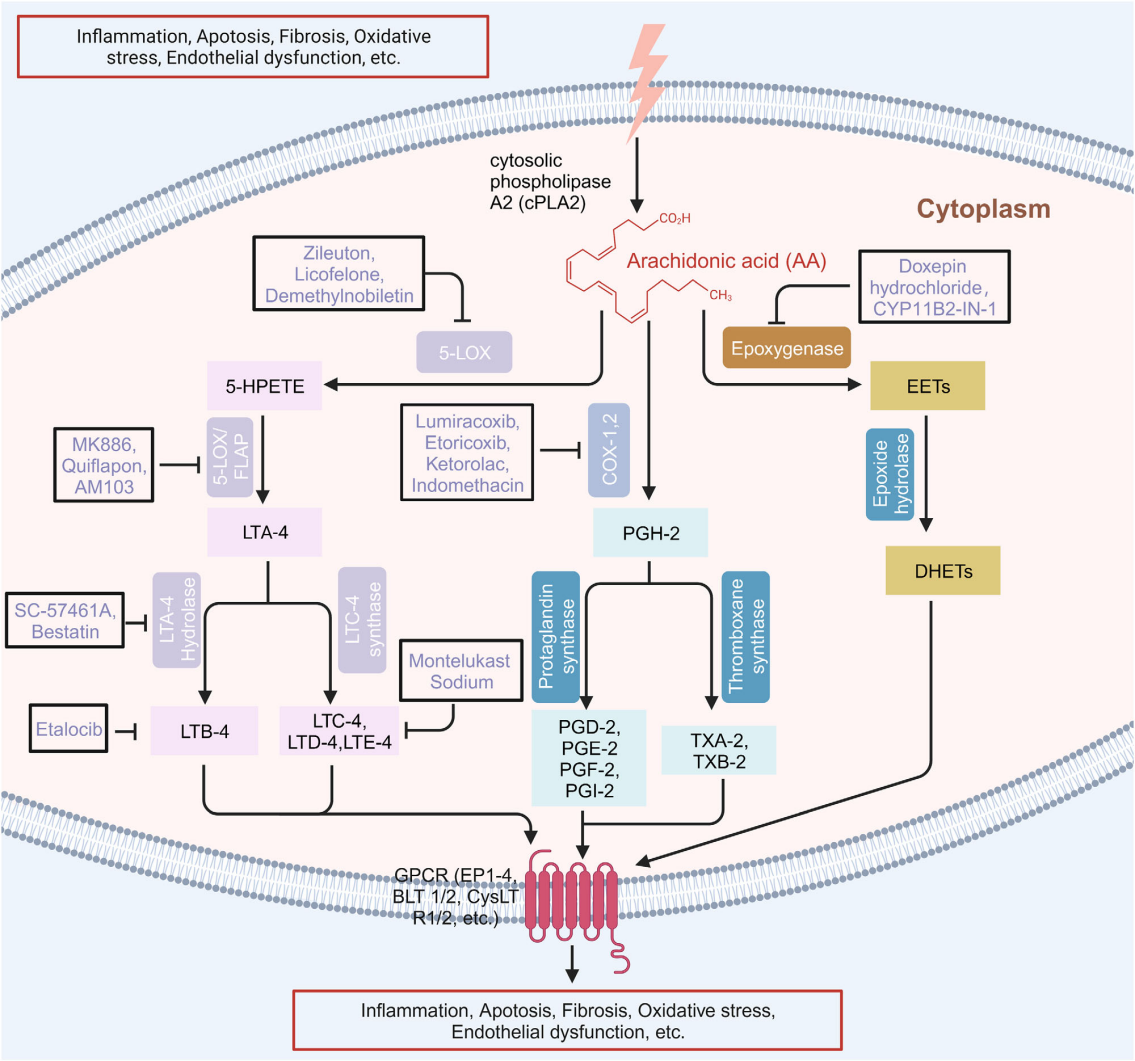
Target inhibitors used to intervene the activation of AA metabolic pathways. AA metabolites play essential roles in regulating inflammation, apoptosis, fibrosis, endothelial dysfunction, and so on. Specific inhibitors, including those used in the clinic and preclinical studies, targeting three AA metabolic pathways are presented here.

Some of them have entered the clinical trial stage for the treatment of tumors or inflammation caused by abnormal AA metabolism and have shown good therapeutic effects and application prospects. We summarized some of the inhibitors targeting AA metabolism‐related enzymes (Table 1 and Figure 6).

**TABLE 1:** Drugs targeting AA metabolism‐related enzymes.

CAS	Inhibitors	Chemical structure	Target enzymes	Mechanism	Clinical trial
111406‐87‐2	Zileuton	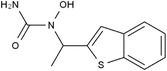	5‐LOX	Catalyze the formation of leukotrienes from arachidonic acid; decreases bronchial mucous secretion and edema	Approved by the US FDA
156897‐06‐2	Licofelone (ML3000)	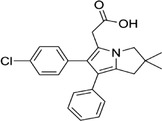	5‐LOX	Inhibit the production of proinflammatory leukotrienes and prostaglandins	Phase 3
2174‐59‐6	Demethylnobiletin	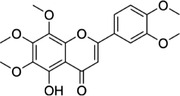	5‐LOX	Anti‐inflammatory activities by inhibiting leukotriene B4 (LTB4) formation in rat neutrophil and elastase release in human neutrophils	N/A
118414‐82‐7	MK886	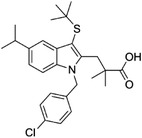	FLAP	Inhibit leukotriene biosynthesis; inhibits 5‐lipoxygenase‐activating protein (FLAP)	N/A
136668‐42‐3	Quiflapon	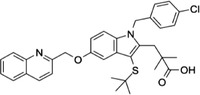	FLAP	Causes cell apoptosis	N/A
1147872‐22‐7	AM103	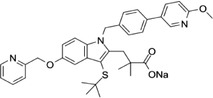	FLAP	Potentially treat asthma and cardiovascular disease by preventing the synthesis of LT	Phase 1
423169‐68‐0	SC‐57461A	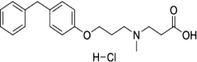	LTA‐4	A potent and specific leukotriene A4 hydrolase (LTA4H) inhibitor	N/A
58970‐76‐6	Bestatin	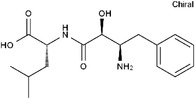	LTA‐4	Inhibit the proliferation of all human leukemia cell lines except KG1	Phase 3
161172‐51‐6	Etalocib	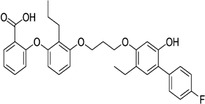	LTB‐4	Block the activation of human neutrophils	Phase 2
151767‐02‐1	Montelukast Sodium	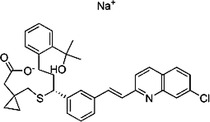	LTD‐4	Promote macroautophagy	Approved by the US FDA
1229‐29‐4	Doxepin hydrochloride	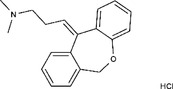	CYP‐450	An orally available tricyclic antidepressant	Approved by the US FDA
1356479‐78‐1	CYP11B2‐IN‐1	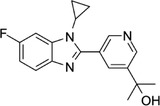	CYP11B2	Dose‐dependently decrease the efficacy of aldosterone without affecting the cortisol levels in rhesus pharmacodynamic models	N/A
220991‐20‐8	Lumiracoxib	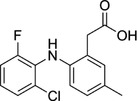	COX‐2	A nonselective nonsteroidal anti‐inflammatory reagent with anti‐inflammatory and antipyretic activity	Phase 4
202409‐33‐4	Etoricoxib	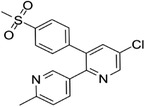	COX‐2	A synthetic nonsteroidal anti‐inflammatory drug (NSAID) with antipyretic, analgesic Potentially antitumor properties	Phase 4
74103‐06‐3	Ketorolac	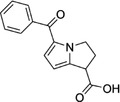	COX‐1/2	A nonsteroidal anti‐inflammatory agent	Approved by the US FDA
53‐86‐1	Indomethacin	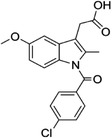	COX‐1/2	Commonly used to reduce fever, pain, stiffness, and swelling	Approved by the US FDA

N/A, not available.

The clinical trial information was obtained from the US FDA (https://www.fda.gov) and DrugBank Online (https://go.drugbank.com). The functions or mechanisms of the above inhibitors were obtained from three different sources (1, https://www.selleck.cn; 2, https://www.medchemexpress.cn; 3, https://www.abmole.cn), and they provided citations, mechanism, and targets for all inhibitors. The structural formulas in this table were drawn using the professional software KingDraw (http://kingdraw.cn/).

### 3.1 Downstream genes of AA metabolism are potential therapeutic targets

Given the nonspecific targeting of some AA‐metabolizing enzyme inhibitors, the downstream genes activated by AA are potential therapeutic targets. In the postirradiation regeneration process, AA positively regulates the proliferation of intestinal epithelial cells and negatively regulates the differentiation of intestinal epithelial cells by upregulating the expression of achaete‐scute family BHLH transcription factor 2 (Ascl2) and activating the WNT signaling pathway. Therefore, functional regulation therapy targeting AA activation of Ascl2 can be considered a potential therapy for radiation damage repair and tissue regeneration.^281^ Myocardial ischemia‒reperfusion (MIR) injury is the main cause of poor revascularization outcomes after myocardial ischemia. ALOX12 is a new upstream regulator of AMPK in the post‐MIR remodeled heart and can be used as a conservative therapeutic target for the treatment of myocardial reperfusion injury.^282^

Tumor cells need to synthesize fatty acids to achieve immune escape and immunotherapeutic resistance, and AA can be used as an antitumor agent in cancer cells with low basic fatty acid synthase levels.^283^, ^284^ Among them, inhibitors of the AA metabolic pathway, especially COX inhibitors, have attracted great attention as promising antitumor drugs.^285^, ^286^, ^287^, ^288^, ^289^, ^290^, ^291^ In addition, specific inhibitors targeting AA and its metabolism‐related enzymes will be potential drug combinations in tumor chemotherapy.^292^, ^293^, ^294^, ^295^ AA is also an ideal target for anti‐inflammatory therapy.^296^, ^297^, ^298^ fatty acid binding protein 3 (FABP3) is highly expressed in the brain, and a FABP3 ligand targeting FABP3 has been identified as a potential therapeutic agent to inhibit αSyn aggregation in vivo.^299^

Other studies have revealed that PIK3CA activates cPLA2 to lead to the overproduction of AA and eicosanoid, and a fatty acid‐rich diet limits the efficacy of cPLA2 inhibitors because PIK3CA mutant tumors may rely on the uptake of extracellular fatty acids to compensate for the loss of AA. Adopting a diet free of meat and dairy products (the main sources of AA) can significantly improve the sensitivity of cPLA2 inhibitors and help to restore tumor immunogenicity, which provides a new method for future clinical trials.^300^

Overexpressed CYP4F2 in non‐small cell lung cancer promotes AA metabolism, which leads to CD8+ T‐cell infiltration and immunosuppression. Therefore, CYP4F2 can be used as a new target to improve the therapeutic efficacy of anti‐programmed death receptor‐1 (PD‐1) therapy, and the combination of CYP4F inhibitors and PD‐1 inhibitors will be a new combination drug strategy for lung cancer immunotherapy.^293^

Through high‐throughput screening, researchers have identified 2,3‐diarylxanone as a potential inhibitor of the AA metabolic pathway that is involved in combination therapy of cancer.^301^ Oxylipins derived from AA have been implicated in the development of colorectal adenomas and colorectal cancer.^302^

### 3.2 Targeting the enzymes involved in AA metabolism can be a therapeutic strategy

Targeting enzymes involved in AA metabolism can also improve radiotherapy.^303^ Long‐chain acyl‐coenzyme A synthases (ACSLs) are responsible for activating long‐chain FAs and are frequently deregulated in cancers.^304^ Several studies suggest that ACSL4 can be used as a biomarker and mediator for the invasive breast cancer phenotype.^305^ The ACSL4 levels correlate positively with the most aggressive quadruple negative breast cancer (QNBC). Furthermore, the induced ACSL4 expression increased cell growth, invasion and resistance to hormones.^136^, ^306^, ^307^ ACSL4 promotes drug resistance in breast cancer cell lines by regulating the expression of energy‐dependent transporters. When the ACSL4 inhibitor triacsin C was used in coordination with chemotherapy drugs, the proliferation of MDA‐MB‐231 wild‐type cells was significantly inhibited. ACSL4 may be regarded as a novel therapeutic target regulating the expression of transporters involved in anticancer drug resistance through the mTOR pathway to restore drug sensitivity in tumors with poor prognosis for disease‐free and overall survival.^308^ Another study showed that high expression of ACSL5 was associated with a better prognosis. The expression levels of ACSL1, ACSL4, and ACSL5 are regulated by the estrogen receptor signaling pathway, and ACSL5 is a potential novel biomarker to predict the prognosis of breast cancer patients.^309^

Abnormal AA metabolism can lead to the development of diseases by activating various signaling pathways. Therefore, targeting the AA metabolic pathway can be a therapeutic strategy to treat related diseases. In COX2‐overexpressing colorectal cancer cells, AA downregulated phosphatase and tensin homolog (PTEN) activity and activated PI3K‐AKT by producing ROS through COX‐2 enzyme‐induced metabolism, promoting the growth of colorectal tumors. COX‐2 inhibitors indirectly promote the expression of PTEN and inhibit the growth of colorectal tumors, making them potential drug targets for colorectal cancer (CRC).^310^

The occurrence and development of chronic diseases are related to AA metabolism. Arteriosclerosis (AS) is a cardiovascular disease that seriously endangers human health. Recent studies have confirmed that AA metabolites are closely related to the occurrence and development of AS. A large body of evidence has shown that dietary supplements comprising omega‐6 polyunsaturated fatty acids, including AA, can increase the distribution of blood lipids and lipoproteins and thus reduce the risk of coronary heart disease.

With the development of natural medicine, targeting natural products of the AA pathway will be a new direction for drug screening. Active ingredients in natural products inhibit the activity of key enzymes involved in AA metabolism, and targeting enzymes involved in AA metabolism can also improve therapeutic efficacy.^311^, ^312^, ^313^, ^314^, ^315^

## 4 CONCLUSION AND PROSPECTS

AA is an important polyunsaturated fatty acid that is the precursor for the synthesis of various bioactive substances in the human body, including prostaglandins, leukotrienes, and platelet‐activating factors. These substances play important physiological roles in the human body, such as regulating immune responses, maintaining vascular stability, and promoting platelet aggregation. Ultrahigh levels of AA in serum or plasma could affect platelet production, thus leading to thrombocytopenia^316^, are considered a risk factor for nonalcoholic fatty liver and liver cirrhosis^317^ and promote cancer cell proliferation in breast cancer.^318^ Low AA levels affects sleep,^319^ elevated blood lipids,^320^ and fetal brain dysplasia.^321^ Therefore, AA metabolism plays an important role in human health.

The abnormal metabolism of AA is closely related to the occurrence and development of many diseases. For example, the metabolic disorder of AA is related to cardiovascular disease, inflammatory bowel disease, asthma, and other diseases. In addition, abnormal metabolism of AA is related to the occurrence and development of metabolic diseases such as obesity and diabetes. Therefore, in‐depth study of AA metabolism is of great significance for the prevention and treatment of these diseases.

There is no doubt that research on the relationship between AA metabolic pathways and human health will be investigated further. Gene editing technology can be used to study the function of genes related to AA metabolism and reveal their role in the occurrence and development of diseases. In addition, the metabolome is a key route to understand the biological functions of related metabolites. The establishment of new methodologies, including real‐time and cell‐specific lipidomic profiling, provides an opportunity to gain a better understanding of the complexity of AA metabolism and may help to improve current treatment strategies and establish new approaches to regulate tissue development and combat diseases. We believe that there will be more specific drugs targeting the AA metabolic pathway. At the same time, combining chemotherapeutic agents or immunotherapies to exploit synergistic therapeutics will be an important trend in the future.

## AUTHOR CONTRIBUTIONS

Q. M. provided the main writing ideas. Y. Z., Y. L., and J. S. wrote different sections of the manuscript. Y. Z. prepared all the figures. W. Z. and Z. G. provided important guidance for this manuscript. All authors have made direct and intellectual contributions to the manuscript and approved the final version.

## CONFLICT OF INTEREST STATEMENT

The authors declare that they have no competing interests.

## ETHICS STATEMENT

Not applicable.

## Data Availability

Not applicable.
